# Patterns and trends of mortality in patients with arrhythmia and cerebrovascular disease in the United States: a nationwide analysis of older adults

**DOI:** 10.1038/s41598-026-64412-z

**Published:** 2026-07-30

**Authors:** Mohamed Fawzi Hemida, Alaa Eldeeb, Maryam Saghir, Alyaa Ahmed Ibrahim, Sahil Jairamani, Abdullah Farahat Elbanna, Amr Ibrahim, Bassam Hegazy, Eshal Saghir, Mohamed Zabady, Walid Bechibchi, Mostafa Mohamed Abdelmoneam Elsawy, Mohamed Alatfawy, Mohamed Elfeki, Karim Othman Abdelnaim, Mustafa Al-jarshawi

**Affiliations:** 1 Faculty of Medicine, Alexandria University, Alexandria, Egypt; 2 Jinnah Sindh Medical University, Karachi, Pakistan; 3 Liaquat University of Medical and Health Sciences, Jamshoro, Pakistan; 4 Department of Internal Medicine, Vanderbilt University Medical Center, Nashville, TN USA; 5 Faculty of Medicine, Monoufia University, Monoufia, Egypt; 6 Dow University of Health Sciences, Karachi, Pakistan; 7 Faculty of Medicine, University of Constantine 3, Salah Boubnider, Constantine, Algeria; 8 Faculty of Medicine, October 6 University, Giza, Egypt; 9 Faculty of Medicine, Kafr Elsheikh University, Kafr El-Shaikh, Egypt; 10 Hamad Medical Corporation, Doha, Qatar; 11 Faculty of Medicine, Ain Shams University, Cairo, Egypt; 12 NIHR Academy, National Institute for Health & Care Research, Leeds, UK; 13 Keele Cardiovascular Research Group, Faculty of Medicine & Health Sciences, Keele University, Newcastle, UK; 14 Faculty of Biology, Medicine and Health, School of Health Sciences, Division of Informatics, Imaging and Data, University of Manchester, Manchester, UK; 15 Cardiology Department, Royal Stoke University Hospitals, University Hospitals of North Midlands NHS Trust, Stoke-on-Trent, UK; 16 Institute of Health Sciences Education, Queen Mary University of London, London, UK

**Keywords:** Arrhythmias, Cerebrovascular disease, Stroke, Mortality trends, Aging population, Cardiovascular epidemiology, Comorbidity, Disparities, Cardiology, Diseases, Health care, Medical research, Risk factors

## Abstract

Arrhythmias and cerebrovascular disease commonly coexist in older adults and are associated with increased mortality risk. However, population-level trends and demographic disparities in related deaths remain insufficiently characterized, necessitating large-scale analyses to better inform prevention, risk stratification, and healthcare planning. Data from CDC WONDER (1999–2023) identified U.S. mortality rates in adults aged (≥ 65 years) with arrhythmia (ICD-10: I47-I49) and cerebrovascular disease (ICD-10: I60-I69) among U.S. adults aged 65 and older. Age-adjusted mortality rates (AAMRs) per 100,000 were stratified by sex, race, urbanization and regions. Trends were analyzed using Joinpoint regression to estimate annual percent change (APC) and average annual percent change (AAPC). Between 1999 and 2023, a total of 733,476 deaths among older adults (≥ 65 years) were attributed to arrhythmias and cerebrovascular disease. The AAMRs exhibited an upward trend from 72.14 in 1999 to 74.62 in 2023 (AAPC: 0.055, 95% CI − 0.62 to 0.74; *p* 0.87) with a significant rise in AAMR from 65.51 in 2018 to 80.44 in 2021 (APC: 6.65; *p *0.01). Men had higher AAMR than women during 1999–2023 with (73.97 to 81.27 vs. 70.22 to 69.2). NH White individuals had the highest AAMR in 2021 (87.43) with a total (631,483 deaths). The South recorded the most deaths (251,478) with a significant decline in AAMR from (70.36) in 2002 to (58.54) in 2008 (APC: − 2.89, 95% CI − 3.85 to − 1.92; *p *0.004). Non-metropolitan areas had higher AAMR compared to metropolitan areas (73.62 vs. 64.71). For the place of death, the majority occurred in inpatient medical facilities (38.73%, 284,079 deaths). Mortality related to arrhythmias and cerebrovascular disease has increased in recent years among older adults. While women accounted for more deaths, men consistently had higher AAMRs. Higher mortality burden was also observed among NH White individuals, residents of the South, and urban populations, with most deaths occurring in inpatient medical facilities, highlighting the need for targeted interventions.

**Supplementary Information:**

The online version contains supplementary material available at 10.1038/s41598-026-64412-z.

## Introduction

Cerebrovascular diseases comprise a spectrum of ischemic and hemorrhagic vascular disorders of the brain, with stroke representing the most common clinical manifestation, which remains a major global cause of mortality and disability^1–3^. Most strokes are ischemic in nature, while the remainder consist of intracerebral and subarachnoid hemorrhages^4^. Globally, stroke represents a substantial public health burden, affecting around 101 million individuals and accounting for approximately 6.55 million deaths in 2019 alone^5^. In the United States, stroke is the fifth leading cause of death, with an estimated 9.4 million adults with a history of stroke^6,7^. In addition to its health impact, stroke represents a considerable economic burden at the healthcare system level^8^.

Cardiac arrhythmias are disorders of the heart’s electrical system that alter normal rhythm, leading to abnormally fast, slow, or irregular rhythm^9^. Atrial fibrillation (AF) represents the most common cardiac rhythm disturbance and has been consistently identified as an independent risk factor for cerebrovascular disease^10,11^. AF-related strokes are generally associated with greater clinical severity and increased rates of morbidity and mortality^12,13^. Atrial fibrillation currently affects an estimated 37.6 million people worldwide, with its global burden rising by roughly one-third over the past 20 years. In the United States alone, the affected population is expected to reach between 6 and 12 million by 2050^14^. Importantly, AF prevalence increases markedly with advancing age, affecting approximately 6% of adults older than 65 years, with nearly 70% of all individuals with AF being between 65 and 85 years of age^15^. Consistent with this pattern, the burden of both cerebrovascular disease and arrhythmias increases with advancing age, with the highest rates observed among adults aged 65 years and older^15,16^.

Prior studies have evaluated temporal patterns and demographic disparities in arrhythmia-related and cerebrovascular mortality separately^17,18^. Although the clinical association between cardiac arrhythmias, particularly atrial fibrillation, and cerebrovascular disease is well established, population-level analyses examining long-term mortality trends and demographic disparities among descendants with coexisting arrhythmias and cerebrovascular disease remain limited. Therefore, this study examined 25-year temporal trends in arrhythmia-associated cerebrovascular mortality among U.S. adults aged 65 years and older using data from the Centers for Disease Control and Prevention Wide-Ranging Online Data for Epidemiologic Research (CDC WONDER) database from 1999 to 2023. Ultimately, this analysis aims to identify high-risk populations and inform targeted strategies to reduce mortality associated with arrhythmia-associated cerebrovascular disease.

## Methods

### Study design and population

Our retrospective analysis was conducted using death certificate data retrieved from the CDC WONDER database to analyze data for older adults aged 65 years and older between 1999 and 2023 and assess cerebrovascular disease- and arrhythmia-related mortality in the United States. This age cutoff has been used to define older adults in similar studies^19^. We used the International Statistical Classification of Diseases and Related Health Problems-10th Revision (ICD-10) as follows: I60–I69 for cerebrovascular disease and I47–I49 for arrhythmias. Deaths were included if at least one ICD-10 code from cerebrovascular disease (I60–I69) AND at least one ICD-10 code from cardiac arrhythmias (I47–I49) were simultaneously listed anywhere on the same death certificate, indicating co-occurrence as either an underlying or contributing cause of death. This comprehensive approach ensures the capture of all deaths where cerebrovascular disease and arrhythmias played documented roles, regardless of their position on the death certificate. The study was conducted and reported in accordance with the STROBE (Strengthening the Reporting of Observational Studies in Epidemiology) guidelines^20^. A completed STROBE checklist is provided in Online Appendix 1.

### Data abstraction

Data on population size, year, number of deaths, and demographic characteristics, including sex, race, and geographic region, were extracted. Place of death was categorized into medical facilities, hospice, home and nursing home/long-term care facilities. The National Center for Health Statistics Urban–Rural Classification Scheme was used to assess the population by urban counties per the 2013 U.S. census classification^21^. It is important to note that urban–rural data were consistently available and analyzed only for the period 1999–2020 due to historical limitations in CDC WONDER stratifications. Regions were stratified into Northeast, Midwest, South, and West according to the U.S. Census Bureau definitions.

### Statistical analysis

Age-adjusted mortality rates (AAMRs) per 100,000 population from 1999–2023 were calculated by year, sex, race, region, and urban–rural status with 95% confidence intervals, using the 2000 U.S. standard population^22^. This standardization approach has been applied in prior studies with a similar design^23^. The Joinpoint Regression Program (Joinpoint V 5.4.0.0, National Cancer Institute) was used to estimate the average annual percent change (AAPC) and annual percent change (APC) with 95% confidence intervals to quantify temporal trends in cerebrovascular disease and arrhythmia-related mortality. Joinpoint regression is a segmented log-linear modeling technique used to identify significant changes in trends (join points) over time. The optimal number of join points was determined using permutation tests to ensure best model fit^24^. A *p *value < 0.05 was considered statistically significant.

## Results

A total of 733,476 deaths among older adults (≥ 65 years) were identified between 1999 and 2023. Place-of-death information was available for 733,471 (99.99%) deaths and was missing for 5 records. Among the 733,471 deaths with available place-of-death data, the majority occurred in inpatient medical facilities, accounting for 284,079 deaths (38.73%). Nursing homes or long-term care settings were the second most common location, with 217,871 deaths (29.70%), reflecting the prolonged disease course and increased care needs in older patients. Decedents’ homes followed with 130,559 deaths (17.80%), indicating that a substantial proportion either experienced out-of-hospital events or opted for end-of-life care at home. Hospice facilities accounted for 44,782 deaths (6.10%), underscoring the role of palliative care. Outpatient or emergency departments comprised 24,759 deaths (3.37%), while other locations accounted for 28,186 deaths (3.84%). Deaths declared on arrival totaled 1607 (0.22%), and those occurring in medical facilities with unknown status numbered 426 (0.06%). A small number of deaths occurred in entirely unknown locations (1202; 0.16%) (Supplemental Tables 1 and 2).

### Overall trends

**Fig. 1:**
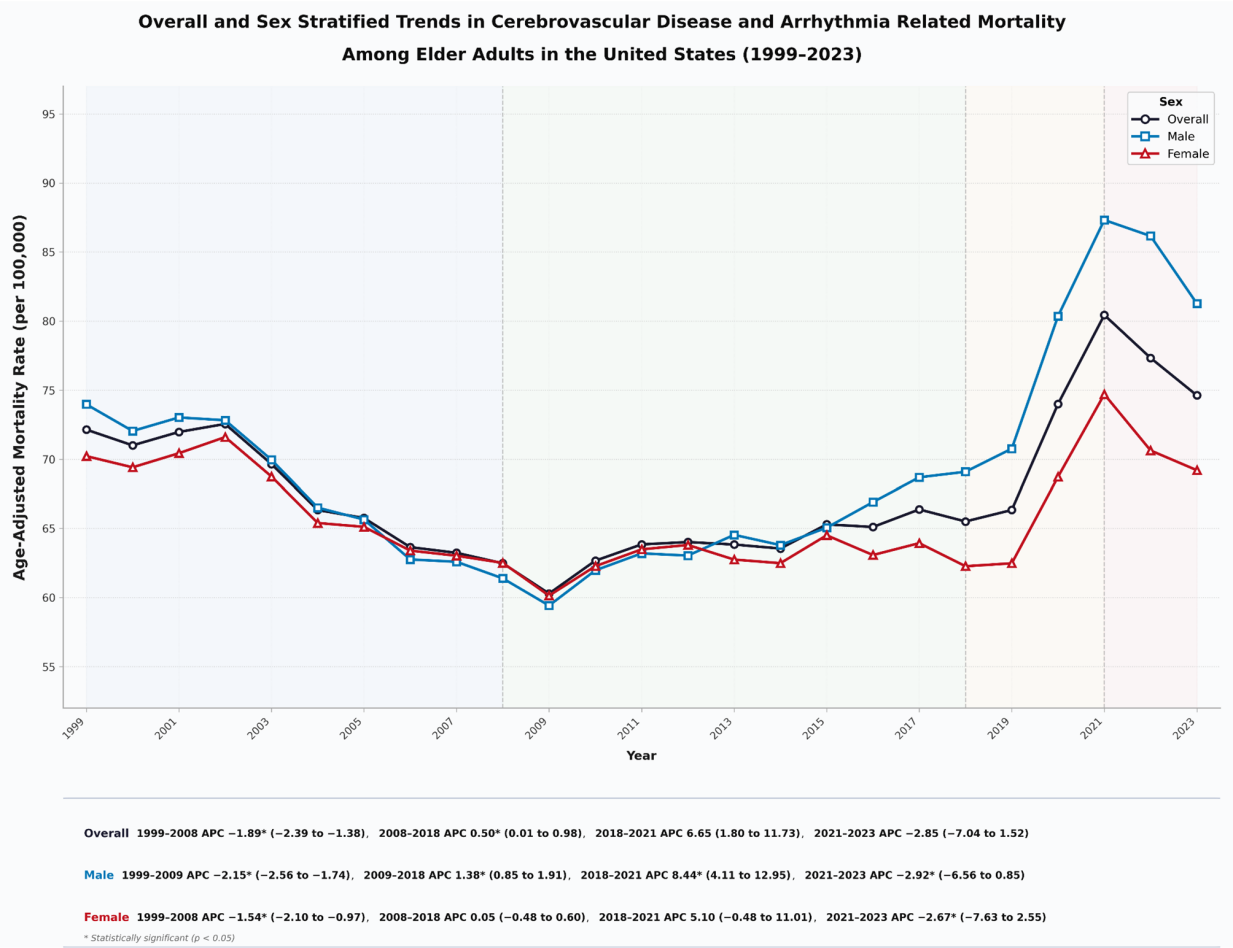
Overall and sex-specific trends in cerebrovascular disease– and arrhythmia-related mortality among older adults in the United States, 1999–2023.

The AAMR in older adults increased non-significantly from 72.14 in 1999 to 74.62 in 2023, with an AAPC of 0.055 (95% CI − 0.62 to 0.74; *p *0.87) (Fig. 1; Supplemental Tables 1, 3, 4).

### Sex-stratified trends

Women experienced an overall higher number of Arrhythmia-related deaths compared to men (females: 443,233 vs. males: 290,243). Older men consistently had higher overall AAMR throughout the study timeline than older women (men: 69.3 vs. women: 65.8). In males, the AAMR increased non-significantly from 73.97 in 1999 to 81.27 in 2023 (AAPC: 0.37; 95% CI − 0.23 to 0.98; *p *0.22). On the other hand, in females, the AAMR decreased from 70.22 in 1999 to 69.2 in 2023 (AAPC: -0.16; 95% CI − 0.95 to 0.63; *p *0.69). (Fig. 1; Supplemental Tables 1, 3, 4).

### Race-stratified trends

**Fig. 2:**
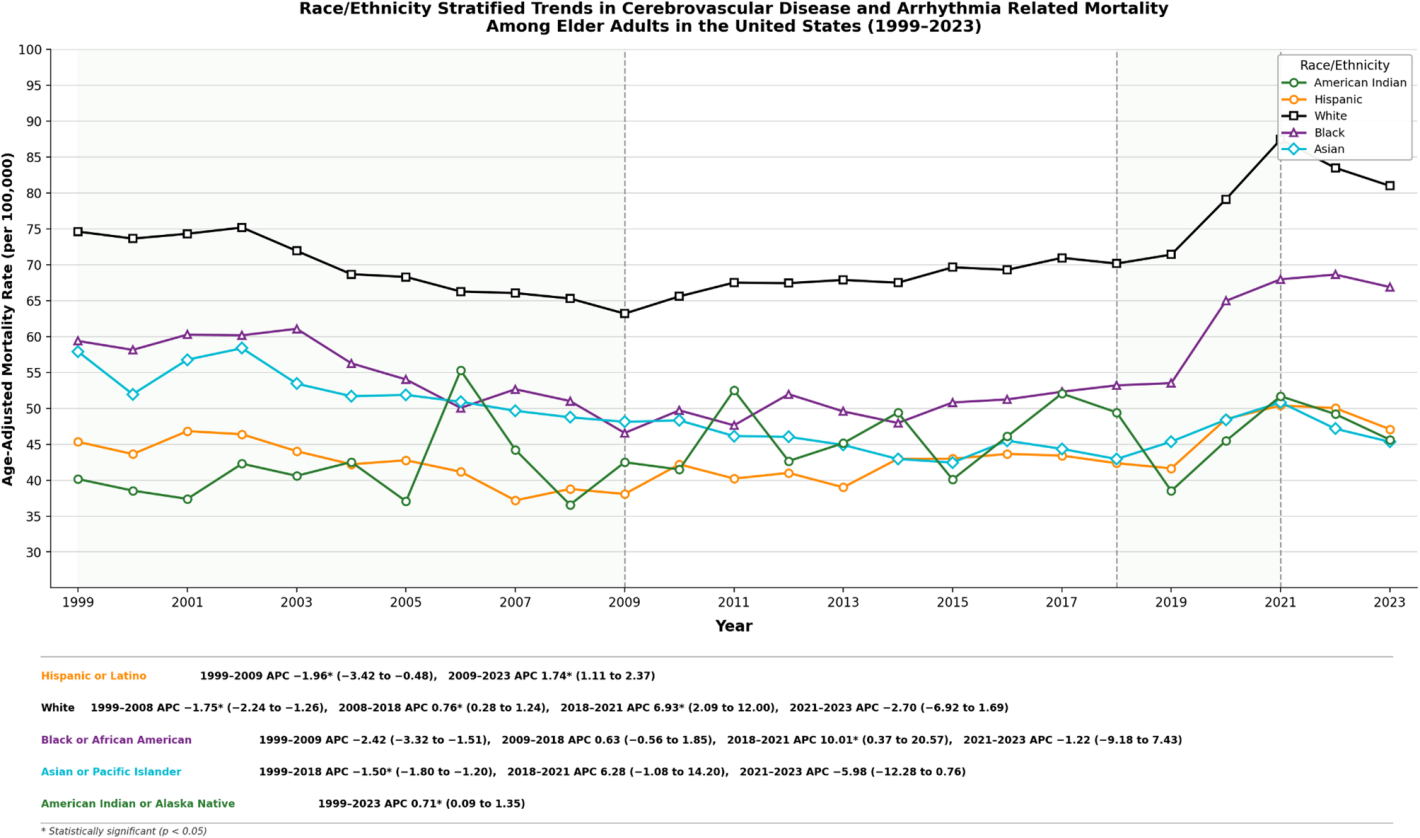
Race/ethnicity-specific trends in cerebrovascular disease– and arrhythmia-related mortality among older adults in the United States, 1999–2023.

When stratified by race/ethnicity, the highest AAMR was observed among non-Hispanic (NH) White individuals (631,483 deaths) (AAMR: 87.43), followed by NH Black, NH Asian, NH American Indian, and Hispanic individuals. Overall, the AAMR decreased nonsignificantly for NH Asian from 57.9 to 45.34 (AAPC: − 0.95; 95% CI − 1.95 to 0.063; *p *0.07). The AAMR increased nonsignificantly for NH Black from 59.38 to 66.89 (AAPC: 0.31; 95% CI − 1.03 to 1.66; *p *0.65), and for NH White from 74.61 to 80.98 (AAPC: 0.26; 95% CI − 0.41 to 0.94; *p *0.45), and Hispanic populations from 45.34 to 47.09 (AAPC: 0.14; 95% CI − 0.49 to 0.86; *p *0.60), and significantly for NH American Indian from 40.15 to 74.86 (AAPC: 0.72; 95% CI 0.09 to 1.35; *p *0.03) between 1999 and 2023. (Fig. 2; Supplemental Tables 3, 5).

### State‑based differences

Between 1999 and 2020, AAMR varied significantly among states, ranging from 40.1 in Nevada to 119.01 in Vermont. However, between 2021 and 2023, the AAMRs were considerably higher, ranging from 40.43 in Connecticut to 138.53 in Oregon (Supplemental Table 6).

### AAMRs stratified by census region

**Fig. 3:**
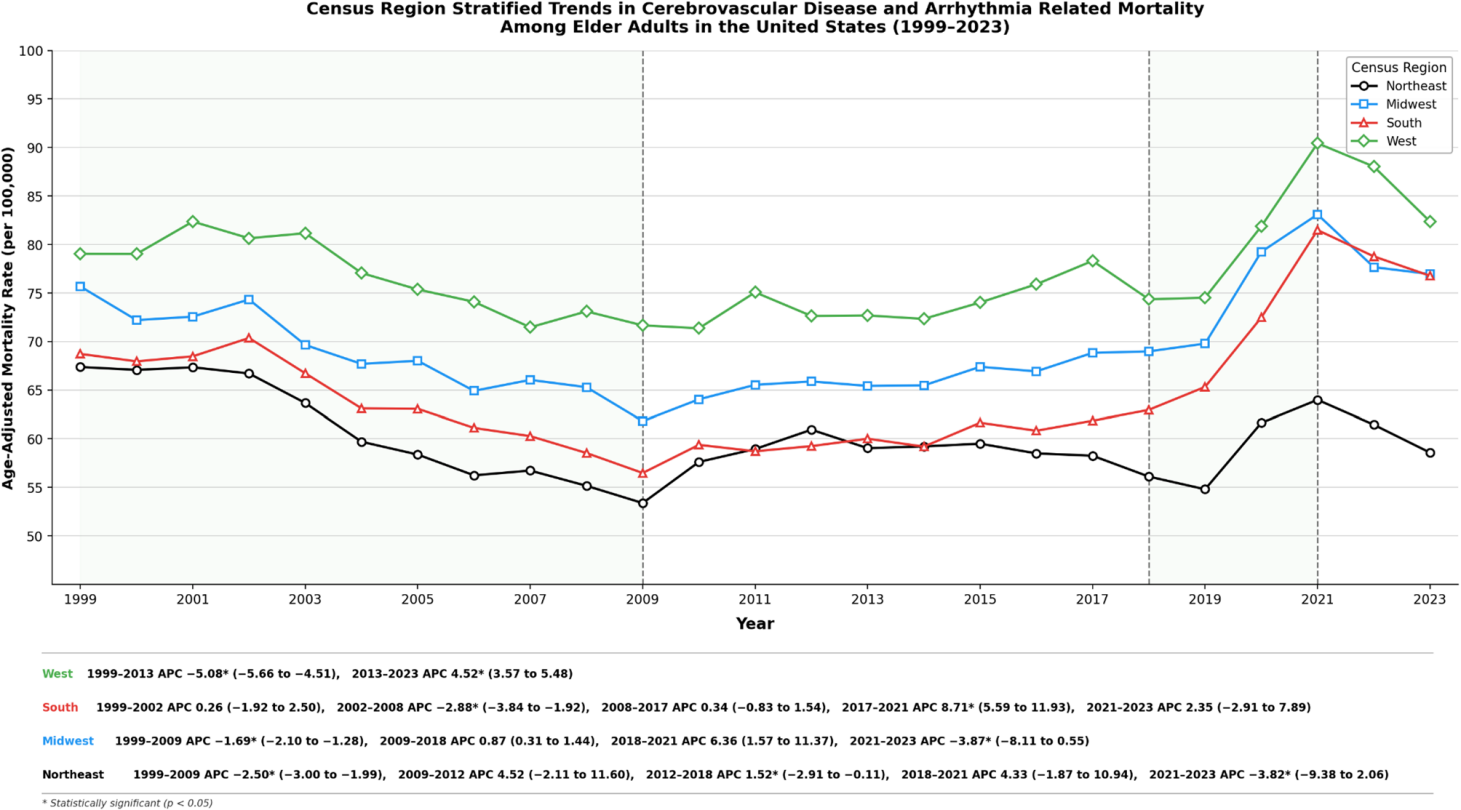
Census region–specific trends in cerebrovascular disease– and arrhythmia-related mortality among older adults in the United States, 1999–2023.

Differences in AAMRs and the number of deaths were observed across the four regions. The South (overall AAMR: 64.94) had the highest number of deaths at 251,478, followed by the West (overall AAMR: 77.15) at 177,115, the Midwest at 172,852 (overall AAMR: 69.73), and the Northeast at 132,031 (overall AAMR: 60.01). In the Northeast, the AAMR decreased from 67.37 to 58.58, with an AAPC of − 0.68 (95% CI − 1.82 to 0.47; *p *0.25). In the Midwest, the overall AAPC from 1999 to 2023 was 0.06 (95% CI − 0.62 to 0.74; *p *0.87), as the AAMR increased from 75.67 to 76.95, indicating no statistically significant trend. The South showed a nonsignificant overall increase in AAMR from 68.72 in 1999 to 76.77 in 2023, with an AAPC of 0.50 (95% CI − 0.09 to 1.08; *p *0.097). From 1999 to 2023, the West region exhibited an overall nonsignificant increase in AAMR from 79.03 to 82.36, with an AAPC of 0.071 (95% CI − 0.88 to 1.03; *p *0.88) (Fig. 3; Supplemental Tables 3, 7).

### Urbanization‑based differences

**Fig. 4:**
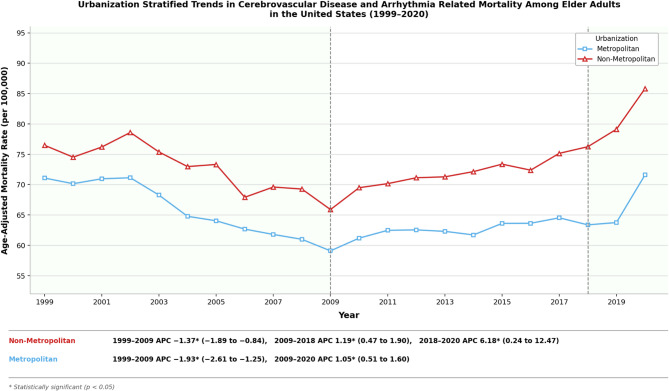
Urbanization-specific trends in cerebrovascular disease– and arrhythmia-related mortality among older adults in the United States, 1999–2020.

From 1999 to 2020, notable urban–rural disparities were observed. In metropolitan areas, the overall AAPC was 0.06 (95% CI − 0.02 to 0.78; *p *0.06), indicating a stable mortality trend, with the AAMR increasing only slightly from 71.08 to 71.60 per 100,000 population. In non-metropolitan areas, the overall AAPC was 0.42 (95% CI − 0.19 to 1.04; *p *0.18), suggesting a nonsignificant upward trend as the AAMR increased from 76.48 to 85.82 (Fig. 4; Supplemental Tables 3, 8).

## Discussion

In this retrospective analysis of 733,476 deaths among older adults (≥ 65 years) in whom both arrhythmias and cerebrovascular disease were reported as multiple causes of death, overall AAMRs remained broadly stable from 1999 to 2023 despite clear inflection points. Mortality declined significantly through the late 2000s, rose again after 2008, accelerated sharply between 2018 and 2021, and then showed a modest decline by 2023. Most deaths occurred in inpatient facilities and nursing homes/long-term care settings, while a substantial minority occurred at home or in hospice. Men had higher AAMRs than women.

The biologic and clinical link between cardiac arrhythmias and cerebrovascular mortality is highly plausible. Although this study encompasses a heterogeneous group of arrhythmias, including supraventricular and ventricular tachyarrhythmias, premature depolarizations, and atrial fibrillation/flutter, AF likely accounts for a substantial proportion of the observed association because it is the arrhythmia most strongly linked to thromboembolic stroke and cerebrovascular mortality in older adults. AF substantially increases thromboembolic stroke risk, and AF-related strokes are generally more disabling and more fatal than non-AF strokes^13,25,26^. The early decline in mortality likely reflects better vascular risk-factor control, broader adoption of stroke prevention strategies in AF, and improvements in antithrombotic treatment over time^27–32^. Landmark randomized trials showed that warfarin reduced stroke risk versus aspirin even in very elderly patients, and later direct oral anticoagulants (DOACs) demonstrated at least noninferior, and in some trials superior, efficacy with lower intracranial bleeding than warfarin^27–31^. Contemporary AF and secondary stroke-prevention guidelines therefore strongly support anticoagulation for eligible high-risk patients, especially older adults, while recognizing the competing bleeding risks and complexity of multimorbidity^32–36^.

Although AF is the arrhythmia most strongly associated with cerebrovascular disease, growing evidence suggests that other cardiac arrhythmias may also contribute to stroke risk. Several studies have demonstrated that non-AF supraventricular arrhythmias, particularly paroxysmal supraventricular tachycardia (PSVT), are associated with an increased risk of ischemic stroke even in the absence of documented AF^37,38^. Proposed mechanisms include shared vascular risk factors, atrial cardiomyopathy, occult AF, and adverse atrial remodeling that may promote thromboembolism before AF becomes clinically apparent^37,38^. In addition, frequent atrial ectopic activity has been linked to a higher long-term risk of stroke and may serve as a marker of underlying structural and electrophysiological abnormalities predisposing to cerebrovascular events^39^. Therefore, the mortality burden observed in this study likely reflects not only the contribution of AF but also the broader impact of other arrhythmias that may directly or indirectly increase cerebrovascular risk.

The renewed increase after 2008, and especially the steep rise from 2018 to 2021, probably reflects several converging forces. First, the aging US population has increased the absolute burden of cardiac arrhythmias, including AF, as well as stroke, frailty, chronic kidney disease, polypharmacy, and competing cardiovascular comorbidities, all of which complicate prevention and treatment in older adults^13,33–35^. Second, undertreatment remains common in older patients who would benefit from anticoagulation, often because of concerns about bleeding, falls, or complexity of care^33–35^. Third, the abrupt rise during 2020–2021 is consistent with the broader reversal in cardiovascular mortality seen during the COVID-19 era^40^. Pandemic-era excess cerebrovascular mortality has already been documented in the United States, and COVID-19–associated ischemic stroke has been linked to worse functional outcomes and markedly higher mortality than non-COVID stroke^41–44^.

Although women accounted for more deaths numerically, men had higher AAMRs across most of the study period. This likely reflects a combination of higher background cardiovascular risk in men and the fact that women live longer and therefore contribute more deaths at older ages. At the same time, the AF literature consistently shows that women with AF may have a higher stroke risk than men once AF is established, particularly in older age groups, and may be less likely to receive rhythm-control strategies or anticoagulation in some settings^45–47^. That framework may help explain why female mortality counts were larger, yet AAMRs remained lower than in men. In other words, the findings fit a pattern in which male patients carry heavier overall age-adjusted cardiovascular mortality, while women with AF may still face important disease-specific disadvantages in recognition, treatment, and stroke sequelae.

The racial findings deserve careful interpretation. NH White individuals had the highest overall AAMRs, but this does not necessarily imply lower vulnerability among other groups. Prior cohort data show that once AF occurs, Black individuals may experience larger excess risks of stroke, heart failure, coronary disease, and mortality than White individuals^48^. Reviews of AF management also document racial and ethnic differences in diagnosis, anticoagulation use, specialist access, and rhythm-control treatment^49^. Accordingly, the lower absolute or AAMRs observed in some racial/ethnic groups may partly reflect differences in ascertainment, competing causes of death, access to care, coding practices, or the well-described “AF paradox,” whereby Black adults have lower recorded AF prevalence despite a higher burden of vascular risk factors and stroke^22,23^. The significant increase among NH American Indian/Alaska Native adults is particularly concerning and may signal worsening exposure to structural barriers, untreated risk factors, or access limitations rather than a purely biological phenomenon.

The strong geographic variation is also consistent with prior U.S. data. Stroke mortality has long shown substantial regional clustering, especially in the South and the so-called Stroke Belt, and rural populations have persistently experienced higher cardiovascular mortality than urban populations^50–53^. The South contributed the largest number of deaths, while nonmetropolitan areas had persistently high and rising mortality, fit this broader literature. These patterns likely reflect differences in socioeconomic deprivation, hypertension and diabetes burden, smoking prevalence, preventive care access, emergency response times, specialist availability, and post-acute rehabilitation resources^51–53^. The interstate spread in mortality rates seen in this analysis further suggests that place-based health-system factors remain highly relevant for older adults with arrhythmia-related cerebrovascular disease.

The distribution of place of death adds another important dimension. The predominance of inpatient and long-term care deaths underscores the high dependency and frailty of this population, while the substantial share of deaths at home and in hospice suggests a growing role for community-based end-of-life care. Stroke survivors and older adults with major rhythm disorders frequently require prolonged nursing support, and stroke is a major contributor to nursing-home dependency^54,55^. Recent stroke palliative-care literature also emphasizes that these patients have significant symptom burden, communication challenges, prognostic uncertainty, and unmet family-support needs, all of which argue for earlier integration of palliative approaches rather than reserving them only for the terminal phase^54–56^. Thus, place-of-death findings are not merely descriptive; they also point to the need for better coordination between acute hospital care, long-term care facilities, hospice, and home-based services.

## Strengths

This study has several strengths. It examines a very large national mortality sample over a long period, which improves precision and allows assessment of temporal inflection points that would be difficult to detect in smaller cohorts. The use of AAMRs strengthens comparability across years and subgroups. In addition, the detailed stratified analyses by sex, race/ethnicity, state, census region, and urbanization provide a broad view of disparities and help generate hypotheses about structural and health-system drivers of mortality. Finally, the inclusion of place-of-death data adds meaningful clinical context by showing where the burden of terminal illness is being experienced, not just how often death occurs.

## Limitations

Several limitations should be acknowledged. First, this is a death-certificate–based retrospective analysis; therefore, causality cannot be inferred. Second, multiple-cause-of-death coding does not establish the temporal sequence between arrhythmia and cerebrovascular disease, nor does it identify the proximate cause of death. Third, cerebrovascular disease was defined using aggregated ICD-10 codes (I60–I69), encompassing both ischemic and hemorrhagic stroke subtypes, which differ in pathophysiology, risk factor profiles, and their associations with arrhythmias—particularly atrial fibrillation, which is more strongly linked to ischemic stroke. As a result, combining these subtypes may introduce misclassification and limit precision in estimating the true burden of arrhythmia-associated cerebrovascular mortality. Moreover, the database does not allow differentiation between stroke subtypes, restricting more granular analyses.

Fourth, the dataset lacks patient-level clinical detail, including arrhythmia subtype. Because ICD-10 codes I47–I49 encompass a heterogeneous group of arrhythmias with varying cerebrovascular risk profiles, the observed mortality trends may not be driven exclusively by atrial fibrillation. Consequently, the findings should be interpreted as reflecting cerebrovascular mortality associated with a broad spectrum of cardiac arrhythmias rather than AF specifically. In addition, the inability to stratify analyses by arrhythmia subtype precludes assessment of subtype-specific mortality patterns. Also, the dataset lacks patient-level clinical detail like anticoagulation status, stroke subtype, rhythm-control therapy, frailty, renal function, socioeconomic status, and healthcare access variables, all of which may confound subgroup trends. Fifth, temporal changes in ICD coding practices, diagnostic intensity, death-certification habits, and awareness of arrhythmias may have influenced observed rates. Sixth, racial and ethnic differences in mortality may partly reflect underdiagnosis or underdocumentation of atrial fibrillation in certain populations. Finally, the pandemic-era increase is temporally suggestive but cannot be directly attributed to COVID-19 without linkage to infection- or hospitalization-level data.

## Conclusion

This large national analysis of older adults demonstrates that mortality related to arrhythmia and cerebrovascular disease remained overall stable from 1999 to 2023 but followed a biphasic pattern, with a significant decline until 2008, a subsequent increase, and a pronounced surge between 2018 and 2021 followed by a slight decline. Most deaths occurred in inpatient and long-term care settings, reflecting advanced disease burden, while a notable proportion occurred at home and hospice. Although women accounted for more deaths, men consistently had higher AAMRs. Higher mortality was observed among NH White individuals, with a concerning rise among NH American Indian populations, as well as higher rates in certain regions and non-metropolitan areas. Future efforts should focus on earlier detection and appropriate management of clinically significant cardiac arrhythmias, optimization of cerebrovascular risk-factor control, equitable implementation of evidence-based stroke-prevention strategies where indicated, improved chronic disease management in older adults, and stronger integration of acute, long-term, and palliative care services.

## Supplementary Information

Below is the link to the electronic supplementary material.

Supplementary Material 1

## Data Availability

The data that support the findings of this study are openly available in CDC‐WONDER at https://wonder.cdc.gov/. Further inquiries can be directed to the corresponding author.
