# Supplementary material for: Patterns and trends of mortality in patients with arrhythmia and cerebrovascular disease in the United States: a nationwide analysis of older adults
Source: Sci Rep. 2026 Jul 30;16:23611. doi: 10.1038/s41598-026-64412-z (PMC13421678; doi:10.1038/s41598-026-64412-z)
Supplement: Supplementary file 1 — Supplementary Material 1 [file 41598_2026_64412_MOESM1_ESM.docx]

**Supplemental Table 1** Cerebrovascular Diseases and Arrhythmia–related Deaths, Stratified by Sex and Race/ Ethnicity, in Older Adults in the United States, 1999 to 2023.

| **Year** | **Overall** | **Female** | **Male** | **NH White** | **NH Black or African American** | **NH Asian or Pacific Islander** | **NH American Indian or Alaska Native** | **Hispanic or Latino** |
| --- | --- | --- | --- | --- | --- | --- | --- | --- |
| 1999 | 24,721 | 15,698 | 9,023 | 22,083 | 1,566 | 369 | 44 | 610 |
| 2000 | 24,663 | 15,747 | 8,916 | 22,046 | 1,547 | 356 | 45 | 617 |
| 2001 | 25,391 | 16,172 | 9,219 | 22,529 | 1,619 | 423 | 46 | 712 |
| 2002 | 25,911 | 16,560 | 9,351 | 22,998 | 1,621 | 457 | 51 | 738 |
| 2003 | 25,257 | 16,104 | 9,153 | 22,307 | 1,671 | 444 | 52 | 738 |
| 2004 | 24,357 | 15,461 | 8,896 | 21,511 | 1,556 | 458 | 55 | 748 |
| 2005 | 24,648 | 15,680 | 8,968 | 21,739 | 1,527 | 506 | 51 | 800 |
| 2006 | 24,364 | 15,529 | 8,835 | 21,478 | 1,446 | 521 | 78 | 815 |
| 2007 | 24,725 | 15,687 | 9,038 | 21,778 | 1,554 | 541 | 63 | 776 |
| 2008 | 24,942 | 15,844 | 9,098 | 21,906 | 1,539 | 556 | 54 | 852 |
| 2009 | 24,557 | 15,514 | 9,043 | 21,528 | 1,452 | 589 | 65 | 880 |
| 2010 | 25,937 | 16,311 | 9,626 | 22,631 | 1,579 | 608 | 64 | 1,022 |
| 2011 | 27,239 | 17,041 | 10,198 | 23,861 | 1,563 | 642 | 86 | 1,059 |
| 2012 | 28,067 | 17,552 | 10,515 | 24,320 | 1,780 | 689 | 78 | 1,143 |
| 2013 | 28,617 | 17,487 | 11,130 | 24,826 | 1,763 | 729 | 84 | 1,164 |
| 2014 | 29,199 | 17,825 | 11,374 | 25,157 | 1,760 | 757 | 101 | 1,362 |
| 2015 | 30,696 | 18,744 | 11,952 | 26,295 | 1,960 | 803 | 90 | 1,457 |
| 2016 | 31,231 | 18,620 | 12,611 | 26,544 | 2,047 | 911 | 107 | 1,562 |
| 2017 | 32,598 | 19,222 | 13,376 | 27,638 | 2,159 | 958 | 125 | 1,653 |
| 2018 | 32,998 | 19,102 | 13,896 | 27,869 | 2,279 | 964 | 131 | 1,700 |
| 2019 | 34,153 | 19,485 | 14,668 | 28,813 | 2,374 | 1,095 | 111 | 1,724 |
| 2020 | 38,798 | 21,675 | 17,123 | 32,247 | 3,013 | 1,238 | 134 | 2,118 |
| 2021 | 39,866 | 22,003 | 17,863 | 33,068 | 3,022 | 1,229 | 141 | 2,165 |
| 2022 | 41,096 | 22,582 | 18,514 | 33,903 | 3,217 | 1,252 | 148 | 2,287 |
| 2023 | 39,445 | 21,588 | 17,857 | 32,408 | 3,201 | 1,234 | 146 | 2,191 |
| Total | 733,476 | 443,233 | 290,243 | 631,483 | 48,815 | 18,329 | 2,150 | 30,893 |

NH, non-Hispanic

**Supplemental Table 2** Cerebrovascular Diseases and Arrhythmia–related Mortality, Stratified by Place of Death in Older Adults in the United States, 1999 to 2023.

| **Place of Death** | **Deaths** |
| --- | --- |
| Medical Facility - Inpatient | 284,079 |
| Medical Facility - Outpatient or ER | 24,759 |
| Medical Facility - Dead on Arrival | 1,607 |
| Medical Facility - Status unknown | 426 |
| Decedent's home | 130,559 |
| Hospice facility | 44,782 |
| Nursing home/long term care | 217,871 |
| Other | 28,186 |
| Place of death unknown | 1,202 |
| **Total** | 733,471 |

**Supplemental Table 3** Annual percent change (APC) of Cerebrovascular Diseases and Arrhythmia–related Age-Adjusted Mortality Rates per 100,000 in Older Adults in the United States, 1999 to 2023.

| **Year Interval** | **APC (95% CI)** |
| --- | --- |
| **Overall** |  |
| 1999–2008 | −1.89 (-2.39 to -1.39) |
| 2008–2018 | 0.50 (0.02 to 0.99) |
| 2018–2021 | 6.65 (1.80 to 11.73) |
| 2021–2023 | −2.85 (-7.05 to 1.53) |
| **Female** |  |
| 1999–2008 | -1.54 (-2.10 to -0.98) |
| 2008–2018 | 0.06 (-0.49 to 0.60) |
| 2018–2021 | 5.11 (-0.48 to 11.01) |
| 2021–2023 | -2.67 (-7.63 to 2.55) |
| **Male** |  |
| 1999–2009 | -2.16 (-2.57 to -1.75) |
| 2009–2018: | 1.38 (0.85 to 1.91) |
| 2018–2021: | 8.45 (4.12 to 12.96) |
| 2021–2023: | -2.93 (-6.57 to 0.85) |
| **NH American Indian or Alaska Native** |  |
| 1999–2023 | 0.72 (0.09 to 1.35) |
| **NH Asian or Pacific Islander** |  |
| 1999–2018 | -1.51 (-1.81 to -1.21) |
| 2018–2021 | 6.29 (-1.08 to 14.21) |
| 2021–2023 | -5.98 (-12.28 to 0.77) |
| **NH Black or African American** |  |
| 1999–2009 | -2.42 (-3.32 to -1.51) |
| 2009–2018 | 0.64 (-0.57 to 1.85) |
| 2018–2021 | 10.01 (0.38 to 20.58) |
| 2021–2023 | -1.23 (-9.19 to 7.43) |
| **NH White** |  |
| 1999–2008 | -1.76 (-2.25 to -1.27) |
| 2008–2018 | 0.76 (0.29 to 1.24) |
| 2018–2021 | 6.94 (2.10 to 12.01) |
| 2021–2023 | -2.71 (-6.92 to 1.70) |
| **Hispanic or Latino** |  |
| 1999–2009 | -1.97 (-3.42 to -0.49) |
| 2009–2023 | 1.74 (1.12 to 2.37) |
| **Metropolitan** |  |
| 1999–2009 | -1.94 (-2.62 to -1.25) |
| 2009–2020 | 1.06 (0.51 to 1.60) |
| **Non-Metropolitan** |  |
| 1999–2009 | -1.37 (-1.89 to -0.84) |
| 2009–2018 | 1.19 (0.48 to 1.91) |
| 2018–2020 | 6.19 (0.25 to 12.48) |
| **Northeast** |  |
| 1999–2009 | -2.50 (-3.01 to -2.00) |
| 2009–2012 | 4.52 (-2.11 to 11.60) |
| 2012–2018 | -1.53 (-2.92 to -0.12) |
| 2018–2021 | 4.34 (-1.88 to 10.95) |
| 2021–2023 | -3.83 (-9.38 to 2.06) |
| **Midwest** |  |
| 1999–2009 | -1.69 (-2.10 to -1.28) |
| 2009–2018 | 0.88 (0.31 to 1.44) |
| 2018–2021 | 6.36 (1.58 to 11.37) |
| 2021–2023 | -3.88 (-8.11 to 0.56) |
| **South** |  |
| 1999–2002 | 0.27 (-1.92 to 2.51) |
| 2002–2008 | –2.89 (-3.85 to -1.92) |
| 2008–2018 | 0.68 (0.30 to 1.06) |
| 2018–2021 | 9.08 (5.32 to 12.97) |
| 2021–2023 | -2.07 (-5.21 to 1.17) |
| **West** |  |
| 1999–2009 | -1.28 (-1.91 to -0.65) |
| 2009–2018 | 0.41 (-0.38 to 1.22) |
| 2018–2021 | 5.84 (-0.86 to 12.99) |
| 2021–2023 | -3.01 (-8.86 to 3.20) |

APC = annual percent change; NH = non-Hispanic.

**Supplemental Table 4** Overall and Sex‐Stratified Cerebrovascular Diseases and Arrhythmia–related Age-Adjusted Mortality Rates per 100,000 in Older Adults in the United States, 1999 to 2023.

| **Year** | **Overall** | **Female** | **Male** |
| --- | --- | --- | --- |
| 1999 | 72.14 (71.24–73.04) | 70.22 (69.12–71.32) | 73.97 (72.41–75.52) |
| 2000 | 71.00 (70.12–71.89) | 69.41 (68.32–70.50) | 72.04 (70.52–73.56) |
| 2001 | 71.97 (71.08–72.85) | 70.43 (69.34–71.52) | 73.02 (71.50–74.53) |
| 2002 | 72.55 (71.67–73.44) | 71.60 (70.51–72.70) | 72.82 (71.32–74.32) |
| 2003 | 69.65 (68.79–70.51) | 68.75 (67.68–69.82) | 69.96 (68.50–71.41) |
| 2004 | 66.34 (65.51–67.17) | 65.40 (64.37–66.44) | 66.51 (65.11–67.91) |
| 2005 | 65.76 (64.94–66.58) | 65.12 (64.09–66.15) | 65.65 (64.28–67.03) |
| 2006 | 63.65 (62.85–64.45) | 63.40 (62.40–64.41) | 62.76 (61.44–64.08) |
| 2007 | 63.24 (62.46–64.03) | 63.03 (62.03–64.02) | 62.59 (61.29–63.89) |
| 2008 | 62.48 (61.71–63.26) | 62.51 (61.53–63.50) | 61.39 (60.12–62.66) |
| 2009 | 60.29 (59.54–61.05) | 60.14 (59.18–61.10) | 59.42 (58.19–60.65) |
| 2010 | 62.67 (61.91–63.44) | 62.27 (61.30–63.24) | 61.97 (60.72–63.21) |
| 2011 | 63.85 (63.09–64.61) | 63.49 (62.52–64.46) | 63.20 (61.97–64.43) |
| 2012 | 64.02 (63.27–64.78) | 63.81 (62.85–64.77) | 63.04 (61.83–64.25) |
| 2013 | 63.84 (63.10–64.59) | 62.75 (61.80–63.70) | 64.54 (63.33–65.74) |
| 2014 | 63.55 (62.82–64.29) | 62.49 (61.56–63.43) | 63.80 (62.61–64.98) |
| 2015 | 65.30 (64.56–66.03) | 64.51 (63.56–65.45) | 65.06 (63.88–66.23) |
| 2016 | 65.11 (64.38–65.84) | 63.08 (62.15–64.00) | 66.91 (65.73–68.08) |
| 2017 | 66.38 (65.66–67.11) | 63.94 (63.02–64.86) | 68.69 (67.52–69.86) |
| 2018 | 65.51 (64.80–66.22) | 62.27 (61.38–63.17) | 69.09 (67.93–70.25) |
| 2019 | 66.34 (65.63–67.05) | 62.48 (61.59–63.36) | 70.76 (69.61–71.91) |
| 2020 | 74.00 (73.26–74.74) | 68.73 (67.81–69.66) | 80.36 (79.14–81.57) |
| 2021 | 80.44 (79.64–81.23) | 74.70 (73.71–75.69) | 87.32 (86.02–88.62) |
| 2022 | 77.33 (76.58–78.08) | 70.63 (69.70–71.55) | 86.16 (84.90–87.42) |
| 2023 | 74.62 (73.88–75.36) | 69.20 (68.28–70.13) | 81.27 (80.05–82.48) |

**Supplemental Table 5** Cerebrovascular Diseases and Arrhythmia–related Age-Adjusted Mortality Rates per 100,000, Stratified by Race/ Ethnicity in Older Adults in the United States, 1999 to 2023.

| **Year** | **NH White** | **NH Black or African American** | **NH Asian or Pacific Islander** | **NH American Indian or Alaska Native** | **Hispanic or Latino** |
| --- | --- | --- | --- | --- | --- |
| 1999 | 74.61 (73.63–75.6) | 59.38 (56.43–62.33) | 57.9 (51.83–63.96) | 40.15 (28.93–54.26) | 45.34 (41.69–48.99) |
| 2000 | 73.64 (72.67–74.61) | 58.14 (55.24–61.05) | 51.96 (46.44–57.48) | 38.54 (28.01–51.74) | 43.63 (40.14–47.12) |
| 2001 | 74.31 (73.34–75.28) | 60.27 (57.32–63.22) | 56.78 (51.25–62.31) | 37.39 (27.17–50.20) | 46.83 (43.33–50.32) |
| 2002 | 75.18 (74.21–76.16) | 60.17 (57.23–63.11) | 58.37 (52.92–63.82) | 42.29 (31.28–55.91) | 46.39 (42.99–49.79) |
| 2003 | 71.92 (70.97–72.86) | 61.08 (58.14–64.02) | 53.43 (48.38–58.49) | 40.60 (30.13–53.52) | 44.03 (40.81–47.26) |
| 2004 | 68.68 (67.76–69.6) | 56.27 (53.46–59.08) | 51.69 (46.89–56.48) | 42.52 (31.76–55.76) | 42.21 (39.14–45.29) |
| 2005 | 68.3 (67.39–69.21) | 54.02 (51.3–56.74) | 51.87 (47.28–56.45) | 37.05 (27.41–48.98) | 42.77 (39.76–45.77) |
| 2006 | 66.27 (65.38–67.16) | 50.06 (47.47–52.65) | 50.91 (46.49–55.33) | 55.28 (43.48–69.30) | 41.17 (38.31–44.03) |
| 2007 | 66.05 (65.17–66.93) | 52.66 (50.03–55.29) | 49.66 (45.44–53.89) | 44.23 (33.84–56.82) | 37.18 (34.54–39.83) |
| 2008 | 65.29 (64.42–66.16) | 51.02 (48.46–53.58) | 48.75 (44.67–52.84) | 36.58 (27.32–47.97) | 38.77 (36.14–41.39) |
| 2009 | 63.19 (62.34–64.04) | 46.57 (44.16–48.98) | 48.12 (44.21–52.04) | 42.49 (32.65–54.36) | 38.07 (35.54–40.6) |
| 2010 | 65.59 (64.73–66.45) | 49.72 (47.26–52.18) | 48.32 (44.46–52.18) | 41.48 (31.80–53.17) | 42.2 (39.6–44.81) |
| 2011 | 67.51 (66.65–68.38) | 47.63 (45.26–50.01) | 46.14 (42.56–49.73) | 52.52 (41.89–65.02) | 40.22 (37.78–42.65) |
| 2012 | 67.43 (66.58–68.29) | 51.97 (49.54–54.4) | 46.03 (42.58–49.48) | 42.66 (33.55–53.47) | 41.01 (38.62–43.4) |
| 2013 | 67.88 (67.03–68.73) | 49.57 (47.23–51.9) | 44.9 (41.62–48.18) | 45.15 (35.91–56.05) | 39 (36.74–41.25) |
| 2014 | 67.49 (66.65–68.33) | 47.96 (45.7–50.22) | 42.94 (39.86–46.02) | 49.41 (39.61–59.22) | 42.98 (40.68–45.27) |
| 2015 | 69.64 (68.79–70.49) | 50.82 (48.55–53.09) | 42.45 (39.49–45.4) | 40.09 (32.07–49.52) | 42.98 (40.76–45.2) |
| 2016 | 69.29 (68.45–70.13) | 51.24 (49–53.49) | 45.49 (42.52–48.47) | 46.09 (37.19–54.99) | 43.65 (41.47–45.83) |
| 2017 | 70.96 (70.11–71.8) | 52.31 (50.07–54.54) | 44.35 (41.52–47.18) | 52.06 (42.80–61.31) | 43.42 (41.31–45.53) |
| 2018 | 70.16 (69.33–70.99) | 53.2 (50.99–55.41) | 42.94 (40.21–45.67) | 49.43 (40.82–58.05) | 42.36 (40.33–44.39) |
| 2019 | 71.41 (70.58–72.23) | 53.51 (51.33–55.69) | 45.36 (42.65–48.06) | 38.50 (31.18–45.81) | 41.64 (39.66–43.62) |
| 2020 | 79.12 (78.25–79.99) | 64.96 (62.6–67.31) | 48.41 (45.69–51.12) | 45.47 (37.65–53.30) | 48.5 (46.42–50.59) |
| 2021 | 87.43 (86.48–88.38) | 67.97 (65.49–70.44) | 50.83 (47.97–53.69) | 51.69 (42.97–60.40) | 50.39 (48.24–52.53) |
| 2022 | 83.49 (82.6–84.38) | 68.63 (66.21–71.04) | 47.16 (44.53–49.79) | 49.20 (41.16–57.23) | 50.03 (47.96–52.1) |
| 2023 | 80.98 (80.1–81.87) | 66.89 (64.52–69.26) | 45.34 (42.8–47.88) | 45.63 (38.13–53.14) | 47.09 (45.1–49.08) |

NH = non-Hispanic.

**Supplemental Table 6** Cerebrovascular Diseases and Arrhythmia–related Age-Adjusted Mortality Rates per 100,000, Stratified by States in Older Adults in the United States, 1999 to 2020.

| **State** | **Age Adjusted Rate (95% CI)** |
| --- | --- |
| Alabama | 61.85 (60.55–63.16) |
| Alaska | 81.28 (75.54–87.03) |
| Arizona | 46.94 (45.97–47.92) |
| Arkansas | 59.88 (58.29–61.46) |
| California | 79.12 (78.57–79.68) |
| Colorado | 69.09 (67.6–70.58) |
| Connecticut | 61.52 (60.15–62.89) |
| Delaware | 62.56 (59.61–65.5) |
| District of Columbia | 59.42 (55.68–63.16) |
| Florida | 44.27 (43.8–44.75) |
| Georgia | 49.67 (48.72–50.62) |
| Hawaii | 64.15 (61.84–66.45) |
| Idaho | 79.97 (77.28–82.67) |
| Illinois | 54.04 (53.3–54.78) |
| Indiana | 70.41 (69.22–71.6) |
| Iowa | 67.08 (65.58–68.59) |
| Kansas | 55.16 (53.65–56.68) |
| Kentucky | 69.33 (67.86–70.8) |
| Louisiana | 45.22 (44.02–46.41) |
| Maine | 74.64 (72.2–77.08) |
| Maryland | 78.17 (76.8–79.54) |
| Massachusetts | 58.33 (57.33–59.33) |
| Michigan | 61.17 (60.3–62.04) |
| Minnesota | 88.29 (86.86–89.72) |
| Mississippi | 55.99 (54.37–57.61) |
| Missouri | 62.78 (61.65–63.9) |
| Montana | 72.23 (69.33–75.13) |
| Nebraska | 74.1 (71.93–76.26) |
| Nevada | 40.1 (38.51–41.69) |
| New Hampshire | 78.74 (76.02–81.46) |
| New Jersey | 63.13 (62.2–64.06) |
| New Mexico | 47.92 (46.13–49.72) |
| New York | 46.26 (45.72–46.79) |
| North Carolina | 76.39 (75.33–77.45) |
| North Dakota | 83.34 (79.78–86.91) |
| Ohio | 82.24 (81.32–83.15) |
| Oklahoma | 72.43 (70.84–74.02) |
| Oregon | 106.44 (104.62–108.26) |
| Pennsylvania | 68.52 (67.78–69.26) |
| Rhode Island | 76.27 (73.51–79.02) |
| South Carolina | 78.44 (76.92–79.97) |
| South Dakota | 71.92 (68.86–74.99) |
| Tennessee | 81.3 (79.98–82.62) |
| Texas | 74.01 (73.29–74.72) |
| Utah | 61.11 (59.02–63.21) |
| Vermont | 119.01 (114.32–123.69) |
| Virginia | 61.56 (60.5–62.62) |
| Washington | 99.68 (98.25–101.1) |
| West Virginia | 83.84 (81.6–86.08) |
| Wisconsin | 70.88 (69.67–72.08) |
| Wyoming | 64.11 (60.08–68.14) |

**Supplemental Table 7** Cerebrovascular Diseases and Arrhythmia–related Age-Adjusted Mortality Rates per 100,000, Stratified by Census Region in Older Adults in the United States, 1999 to 2023.

| **Year** | **Census Region** | **Age Adjusted Rate (95% CI)** |
| --- | --- | --- |
| 1999 | Northeast | 67.37 (65.5–69.23) |
| 2000 | Northeast | 67.07 (65.23–68.92) |
| 2001 | Northeast | 67.34 (65.5–69.18) |
| 2002 | Northeast | 66.7 (64.87–68.52) |
| 2003 | Northeast | 63.66 (61.89–65.42) |
| 2004 | Northeast | 59.7 (57.99–61.4) |
| 2005 | Northeast | 58.38 (56.7–60.05) |
| 2006 | Northeast | 56.24 (54.6–57.87) |
| 2007 | Northeast | 56.73 (55.1–58.36) |
| 2008 | Northeast | 55.16 (53.56–56.75) |
| 2009 | Northeast | 53.38 (51.82–54.94) |
| 2010 | Northeast | 57.62 (56–59.23) |
| 2011 | Northeast | 58.95 (57.33–60.57) |
| 2012 | Northeast | 60.94 (59.31–62.58) |
| 2013 | Northeast | 59.04 (57.44–60.64) |
| 2014 | Northeast | 59.22 (57.63–60.82) |
| 2015 | Northeast | 59.5 (57.91–61.09) |
| 2016 | Northeast | 58.5 (56.93–60.07) |
| 2017 | Northeast | 58.26 (56.72–59.8) |
| 2018 | Northeast | 56.11 (54.61–57.6) |
| 2019 | Northeast | 54.8 (53.33–56.26) |
| 2020 | Northeast | 61.64 (60.09–63.19) |
| 2021 | Northeast | 63.99 (62.38–65.6) |
| 2022 | Northeast | 61.45 (59.92–62.98) |
| 2023 | Northeast | 58.58 (57.08–60.08) |
| 1999 | Midwest | 75.67 (73.81–77.53) |
| 2000 | Midwest | 72.2 (70.38–74.01) |
| 2001 | Midwest | 72.56 (70.76–74.37) |
| 2002 | Midwest | 74.33 (72.51–76.15) |
| 2003 | Midwest | 69.64 (67.89–71.38) |
| 2004 | Midwest | 67.69 (65.98–69.41) |
| 2005 | Midwest | 68.01 (66.3–69.72) |
| 2006 | Midwest | 64.91 (63.25–66.56) |
| 2007 | Midwest | 66.04 (64.38–67.7) |
| 2008 | Midwest | 65.29 (63.65–66.93) |
| 2009 | Midwest | 61.81 (60.22–63.39) |
| 2010 | Midwest | 64.04 (62.44–65.65) |
| 2011 | Midwest | 65.53 (63.92–67.14) |
| 2012 | Midwest | 65.88 (64.29–67.48) |
| 2013 | Midwest | 65.42 (63.84–67.01) |
| 2014 | Midwest | 65.47 (63.9–67.04) |
| 2015 | Midwest | 67.39 (65.81–68.98) |
| 2016 | Midwest | 66.92 (65.35–68.49) |
| 2017 | Midwest | 68.83 (67.26–70.4) |
| 2018 | Midwest | 68.98 (67.42–70.53) |
| 2019 | Midwest | 69.78 (68.22–71.33) |
| 2020 | Midwest | 79.24 (77.59–80.89) |
| 2021 | Midwest | 83.09 (81.35–84.83) |
| 2022 | Midwest | 77.66 (76.03–79.29) |
| 2023 | Midwest | 76.95 (75.32–78.59) |
| 1999 | South | 68.72 (67.23–70.22) |
| 2000 | South | 67.95 (66.48–69.43) |
| 2001 | South | 68.47 (66.99–69.94) |
| 2002 | South | 70.36 (68.87–71.84) |
| 2003 | South | 66.72 (65.29–68.16) |
| 2004 | South | 63.11 (61.73–64.5) |
| 2005 | South | 63.07 (61.7–64.44) |
| 2006 | South | 61.12 (59.79–62.46) |
| 2007 | South | 60.28 (58.97–61.59) |
| 2008 | South | 58.54 (57.26–59.82) |
| 2009 | South | 56.48 (55.24–57.72) |
| 2010 | South | 59.38 (58.11–60.64) |
| 2011 | South | 58.72 (57.49–59.95) |
| 2012 | South | 59.25 (58.04–60.47) |
| 2013 | South | 60.01 (58.8–61.22) |
| 2014 | South | 59.2 (58.01–60.38) |
| 2015 | South | 61.65 (60.46–62.84) |
| 2016 | South | 60.83 (59.66–62) |
| 2017 | South | 61.86 (60.7–63.02) |
| 2018 | South | 62.96 (61.81–64.11) |
| 2019 | South | 65.32 (64.16–66.47) |
| 2020 | South | 72.47 (71.26–73.67) |
| 2021 | South | 81.48 (80.17–82.8) |
| 2022 | South | 78.77 (77.53–80.01) |
| 2023 | South | 76.77 (75.54–77.99) |
| 1999 | West | 79.03 (76.88–81.17) |
| 2000 | West | 79.02 (76.9–81.14) |
| 2001 | West | 82.36 (80.23–84.5) |
| 2002 | West | 80.64 (78.55–82.74) |
| 2003 | West | 81.16 (79.09–83.24) |
| 2004 | West | 77.06 (75.05–79.06) |
| 2005 | West | 75.36 (73.41–77.31) |
| 2006 | West | 74.09 (72.18–76) |
| 2007 | West | 71.46 (69.61–73.31) |
| 2008 | West | 73.1 (71.25–74.94) |
| 2009 | West | 71.66 (69.86–73.47) |
| 2010 | West | 71.36 (69.58–73.15) |
| 2011 | West | 75.07 (73.27–76.86) |
| 2012 | West | 72.63 (70.9–74.37) |
| 2013 | West | 72.68 (70.96–74.4) |
| 2014 | West | 72.33 (70.64–74.01) |
| 2015 | West | 74.02 (72.34–75.7) |
| 2016 | West | 75.9 (74.22–77.58) |
| 2017 | West | 78.31 (76.63–80) |
| 2018 | West | 74.35 (72.73–75.96) |
| 2019 | West | 74.51 (72.92–76.11) |
| 2020 | West | 81.87 (80.22–83.53) |
| 2021 | West | 90.43 (88.64–92.22) |
| 2022 | West | 88.03 (86.33–89.73) |
| 2023 | West | 82.36 (80.71–84) |

**Supplemental Table 8** Cerebrovascular Diseases and Arrhythmia–related Age-Adjusted Mortality Rates per 100,000, Stratified by Urban-Rural Classification in Older Adults in the United States, 1999 to 2020.

| **Year** | **Metropolitan** | **Non-metropolitan** |
| --- | --- | --- |
| 1999 | 71.08 (70.08–72.08) | 76.48 (74.39–78.58) |
| 2000 | 70.15 (69.16–71.13) | 74.52 (72.47–76.58) |
| 2001 | 70.96 (69.98–71.94) | 76.19 (74.12–78.26) |
| 2002 | 71.13 (70.16–72.1) | 78.59 (76.48–80.69) |
| 2003 | 68.28 (67.34–69.23) | 75.38 (73.33–77.43) |
| 2004 | 64.78 (63.87–65.69) | 72.97 (70.96–74.98) |
| 2005 | 64.04 (63.14–64.94) | 73.32 (71.32–75.33) |
| 2006 | 62.67 (61.79–63.55) | 67.91 (65.99–69.83) |
| 2007 | 61.79 (60.93–62.65) | 69.6 (67.67–71.53) |
| 2008 | 60.99 (60.14–61.84) | 69.27 (67.35–71.18) |
| 2009 | 59.09 (58.26–59.92) | 65.89 (64.03–67.75) |
| 2010 | 61.18 (60.34–62.01) | 69.49 (67.59–71.4) |
| 2011 | 62.47 (61.64–63.3) | 70.16 (68.27–72.06) |
| 2012 | 62.54 (61.71–63.36) | 71.13 (69.24–73.02) |
| 2013 | 62.3 (61.48–63.11) | 71.28 (69.41–73.16) |
| 2014 | 61.71 (60.92–62.51) | 72.14 (70.26–74.01) |
| 2015 | 63.61 (62.81–64.41) | 73.37 (71.5–75.25) |
| 2016 | 63.63 (62.83–64.42) | 72.38 (70.52–74.23) |
| 2017 | 64.53 (63.75–65.32) | 75.15 (73.28–77.02) |
| 2018 | 63.37 (62.6–64.14) | 76.24 (74.38–78.1) |
| 2019 | 63.75 (62.99–64.51) | 79.12 (77.24–81) |
| 2020 | 71.6 (70.81–72.4) | 85.82 (83.88–87.77) |

**Appendix 1. STROBE Statement**

Checklist of items that should be included in reports of observational studies

| **Section/Topic** | **Item No** | **Recommendation** | **Reported on Page No** |
| --- | --- | --- | --- |
| **Title and abstract** | 1 | (*a*) Indicate the study’s design with a commonly used term in the title or the abstract | 2,3 |
|  |  | (*b*) Provide in the abstract an informative and balanced summary of what was done and what was found | 2,3 |
| **Introduction** | | | |
| Background/rationale | 2 | Explain the scientific background and rationale for the investigation being reported | 3, 4 |
| Objectives | 3 | State specific objectives, including any prespecified hypotheses | 3, 4 |
| **Methods** | | | |
| Study design | 4 | Present key elements of study design early in the paper | 4, 5 |
| Setting | 5 | Describe the setting, locations, and relevant dates, including periods of recruitment, exposure, follow-up, and data collection | 4, 5 |
| Participants | 6 | (*a*) *Cohort study*—Give the eligibility criteria, and the sources and methods of selection of participants. Describe methods of follow-up  *Case-control study*—Give the eligibility criteria, and the sources and methods of case ascertainment and control selection. Give the rationale for the choice of cases and controls  *Cross-sectional study*—Give the eligibility criteria, and the sources and methods of selection of participants | 4, 5, 6 |
|  |  | (*b*) *Cohort study*—For matched studies, give matching criteria and number of exposed and unexposed  *Case-control study*—For matched studies, give matching criteria and the number of controls per case | 4, 5, 6 |
| Variables | 7 | Clearly define all outcomes, exposures, predictors, potential confounders, and effect modifiers. Give diagnostic criteria, if applicable | 4, 5, 6 |
| Data sources/measurement | 8* | For each variable of interest, give sources of data and details of methods of assessment (measurement). Describe comparability of assessment methods if there is more than one group | 4, 5, 6 |
| Bias | 9 | Describe any efforts to address potential sources of bias | 4, 5, 6 |
| Study size | 10 | Explain how the study size was arrived at | 4, 5, 6 |
| Quantitative variables | 11 | Explain how quantitative variables were handled in the analyses. If applicable, describe which groupings were chosen and why | 4, 5, 6 |
| Statistical methods | 12 | (*a*) Describe all statistical methods, including those used to control for confounding | 4, 5, 6 |
|  |  | (*b*) Describe any methods used to examine subgroups and interactions | 4, 5, 6 |
|  |  | (*c*) Explain how missing data were addressed | 4, 5, 6 |
|  |  | (*d*) *Cohort study*—If applicable, explain how loss to follow-up was addressed  *Case-control study*—If applicable, explain how matching of cases and controls was addressed  *Cross-sectional study*—If applicable, describe analytical methods taking account of sampling strategy | 4, 5, 6 |
|  |  | (*e*) Describe any sensitivity analyses | NA |

| **Section/Topic** | **Item No** | **Recommendation** | **Reported on Page No** |
| --- | --- | --- | --- |
| **Results** | | | |
| Participants | 13* | (a) Report numbers of individuals at each stage of study—eg numbers potentially eligible, examined for eligibility, confirmed eligible, included in the study, completing follow-up, and analysed | 6, 7, 8 |
|  |  | (b) Give reasons for non-participation at each stage | NA |
|  |  | (c) Consider use of a flow diagram | NA |
| Descriptive data | 14* | (a) Give characteristics of study participants (eg demographic, clinical, social) and information on exposures and potential confounders | 6, 7, 8 |
|  |  | (b) Indicate number of participants with missing data for each variable of interest | NA |
|  |  | (c) *Cohort study*—Summarise follow-up time (eg, average and total amount) | NA |
| Outcome data | 15* | *Cohort study*—Report numbers of outcome events or summary measures over time | NA |
|  |  | *Case-control study—*Report numbers in each exposure category, or summary measures of exposure | NA |
|  |  | *Cross-sectional study—*Report numbers of outcome events or summary measures | NA |
| Main results | 16 | (*a*) Give unadjusted estimates and, if applicable, confounder-adjusted estimates and their precision (eg, 95% confidence interval). Make clear which confounders were adjusted for and why they were included | 6, 7, 8, 9 |
|  |  | (*b*) Report category boundaries when continuous variables were categorized | NA |
|  |  | (*c*) If relevant, consider translating estimates of relative risk into absolute risk for a meaningful time period | NA |
| Other analyses | 17 | Report other analyses done—eg analyses of subgroups and interactions, and sensitivity analyses | NA |
| **Discussion** | | | |
| Key results | 18 | Summarise key results with reference to study objectives | 16-18 |
| Limitations | 19 | Discuss limitations of the study, taking into account sources of potential bias or imprecision. Discuss both direction and magnitude of any potential bias | 18 |
| Interpretation | 20 | Give a cautious overall interpretation of results considering objectives, limitations, multiplicity of analyses, results from similar studies, and other relevant evidence | 16-18 |
| Generalisability | 21 | Discuss the generalisability (external validity) of the study results | NA |
| **Other Information** | | | |
| Funding | 22 | Give the source of funding and the role of the funders for the present study and, if applicable, for the original study on which the present article is based | NA |
